# Whole-Genome Sequencing of ST2 *A. baumannii* Causing Bloodstream Infections in COVID-19 Patients

**DOI:** 10.3390/antibiotics11070955

**Published:** 2022-07-15

**Authors:** Sabrina Cherubini, Mariagrazia Perilli, Bernardetta Segatore, Paolo Fazii, Giustino Parruti, Antonella Frattari, Gianfranco Amicosante, Alessandra Piccirilli

**Affiliations:** 1 Department of Biotechnological and Applied Clinical Sciences, University of L’Aquila, 67100 L’Aquila, Italy; sabrina.cherubini@graduate.univaq.it (S.C.); mariagrazia.perilli@univaq.it (M.P.); bernardetta.segatore@univaq.it (B.S.); gianfranco.amicosante@univaq.it (G.A.); 2 Clinical Microbiology and Virology Unit, Spirito Santo Hospital, 65122 Pescara, Italy; paolo.fazii@ausl.pe.it; 3 Infectious Diseases, Spirito Santo Hospital, 65122 Pescara, Italy; giustino.parruti@ausl.pe.it; 4 Intensive Care Unit, Spirito Santo Hospital, 65122 Pescara, Italy; antonella.frattari@ausl.pe.it

**Keywords:** *Acinetobacter baumannii*, COVID-19, WGS

## Abstract

A total of 43 *A. baumannii* strains, isolated from 43 patients affected by severe acute respiratory syndrome coronavirus 2 (SARS-CoV-2) and by bacterial sepsis, were analyzed by antimicrobial susceptibility testing. All strains were resistant to almost three different classes of antibiotics, including carbapenems and colistin. The whole-genome sequencing (WGS) of eight selected *A. baumannii* isolates showed the presence of different insertion sequences (ISs), such as IS*Aba13*, IS*Aba26*, IS*26*, IS*Vsa3*, IS*Ec29*, IS*6100* and IS*17,* giving to *A. baumannii* a high ability to capture and mobilize antibiotic resistance genes. Resistance to carbapenems is mainly mediated by the presence of OXA-23, OXA-66 and OXA-82 oxacillinases belonging to OXA-51-like enzymes. The presence of AmpC cephalosporinase, ADC-25, was identified in all *A. baumannii*. The pathogenicity of *A. baumannii* was exacerbated by the presence of several virulence factors. The multi-locus sequence typing (MLST) analysis showed that all strains belong to sequence type 2 (ST) international clone.

## 1. Introduction

*Acinetobacter baumannii* belongs to a group of nosocomial pathogens, designated by the acronym “ESKAPE” (*Enterococcus faecium*, *Staphylococcus aureus*, *Klebsiella pneumoniae*, *Acinetobacter baumannii*, *Pseudomonas aeruginosa* and *Enterobacter* spp.). These bacteria are the predominant cause of life-threatening nosocomial infections in critically ill and immunocompromised patients worldwide. ESKAPE pathogens have developed resistance mechanisms against almost all antimicrobial agents, including carbapenems, the last line of defense in clinical armamentarium [1,2,3,4]. *A. baumannii* is commonly associated with nosocomial infections including bacteremia, endocarditis, urinary tract infections (UTIs), meningitis, gastrointestinal and skin/wound infections [5,6,7,8]. However, community-acquired *A. baumannii* infections have been described, in particular, in people with comorbidities [9,10,11]. Several studies have reported that respiratory viral infections, such as severe acute respiratory syndrome coronavirus 2 (SARS-CoV-2), predispose patients to bacterial co-infections and secondary infections [12]. Prolonged hospitalization in the intensive care unit (ICU) may be linked with increased possibility of developing bacterial co-infection, especially with multi-drug resistant (MDR) *A. baumannii* in critically ill coronavirus disease 2019 (COVID-19) patients. Generally, antibiotic resistance in *A. baumannii* is due to different mechanisms: (a) inactivation of antibiotics by enzymatic hydrolysis (i.e., β-lactamases), (b) reducing membrane permeability, (c) increasing efflux of antibiotics (i.e., overexpression of drug efflux pumps) and (d) mutations in antibiotic binding targets by genetic insertion sequences. Since 1990, carbapenems have been the mainstay antibiotics in the treatment of *A. baumannii* infections. Currently, carbapenem-resistant *A. baumannii* (CRAb) poses a global threat to human health. CRAb is emerging worldwide, and the majority of these isolates often show MDR or extensive drug resistant (XDR) patterns [13,14]. In 2018, the World Health Organization (WHO) considered *A. baumannii* a priority for the research and development of new antibiotics [3]. The presence of β-lactamases, in particular oxacillinases (OXAs), constitutes the main mechanism of resistance to carbapenems in *A. baumannii*. The main *bla* genes found in *A. baumannii* are represented by the presence of intrinsic chromosomal *bla*_OXA-51-like_ genes and other groups of OXA acquired carbapenemases, including OXA-23-like, OXA-24/40-like, OXA-58-like, and OXA-143 enzymes [15]. In a large number of studies, ISAba1 has been identified in combination with different OXA-β-lactamases, including the *bla*_OXA−51-like_ genes. This insertion sequence is located upstream in the opposite direction of *bla*_OXA−51-like_, acting as a promoter [16]. Among other β-lactamases, class A β-lactamases (e.g., PER-1, VEB-1, CTX-M-1, TEM-1), metallo β-lactamases (e.g., IMP, VIM, NDM) and AmpC cephalosporinases are prevalent in *A. baumannii* [17,18]. *A. baumannii* is also able to promote antibiotic resistance by virulence factors such as outer membrane protein modification, cell envelope factors, enzymes, quorum sensing, biofilm formation, motility and micronutrient acquisition systems [19]. Currently, very few therapeutic agents exist or are in development to treat MDR *A. baumannii* infections. Indeed, the ability of *A. baumannii* to persist and easily survive on biotic and abiotic surfaces and under dry conditions makes its treatment and eradication really complicated [20]. Because of the decrease in susceptibility to a wide range of antibiotics, the treatment options are frequently limited to synergistic use of these agents. Despite its nephrotoxicity, colistin, as monotherapy or in combination with other active compounds, has been used in critically ill patients [21]. Unfortunately, colistin-resistant Acinetobacter strains have rapidly emerged [22,23,24]. In recent times, cefiderocol, a novel siderophore cephalosporin, and durlobactam were approved for the treatment of MDR Gram-negative infections, including *A. baumannii* [25,26,27]. The aim of the present study was to explore, by whole-genome sequencing (WGS), antibiotic resistance genes (ARGs) and virulence factors of *A. baumannii* strains isolated from COVID-19 patients affected by sepsis and admitted to the ICU of Spirito Santo Hospital, Pescara, Central Italy.

## 2. Results

### 2.1. Minimal Inhibitory Concentrations (MICs)

A total of 43 *A. baumannnii* strains, isolated from positive blood cultures, were analyzed against a panel of antibiotics. All the strains showed the same antimicrobial susceptibility profile with a remarkable resistance to amikacin (MIC ≥ 64 mg/L), gentamicin (MIC ≥ 16 mg/L), ciprofloxacin (MIC ≥ 4 mg/L), meropenem (MIC > 8 mg/L), colistin (MIC > 8 mg/L) and trimethoprim–sulfamethoxazole association (MIC > 160 mg/L).

### 2.2. WGS and Multi-Locus Sequence Typing (MLST)

The WGS was performed on eight non-replicative *A. baumannii* strains isolated from patients admitted to ICU in different time periods. By molecular analysis we identified the sequence type (MLST), ARGs, virulence factors and mobile genetic elements (MGEs) harbored in the analyzed genome of *A. baumannii*. The genome size of the eight *A. baumannii* strains ranged from 3.8 to 4.0 Mb, and no plasmids were found. The MLST analysis using the Pasteur scheme revealed that all isolates belonged to the ST2 sequence type (Table 1).

### 2.3. Antimicrobial Resistance Genes (ARGs)

The *A. baumannii* isolates showed a wide variety of antibiotic resistance genes, mobile genetic elements (e.g.*,* IS, sequence insertion) and several virulence factors (Table 1).

#### 2.3.1. β-Lactam Resistance Genes

The *A. baumannii* strains analyzed in this study harbored molecular class C and class D β-lactamases. Overall, *bla*_ADC-25_ and *bla*_OXA-23_ genes were found in all isolates with a co-presence of *bla*_OXA-66_ (four out of eight isolates) or *bla*_OXA-82_ (four out of eight isolates) (Table 1). Both *bla*_OXA-66_ and *bla*_OXA-82_ belong to the constitutive *bla*_OXA-51-type_ genes. The OXA-82 enzyme differs from OXA-66 only by one amino acid residue. The leucine 157 (L157) in OXA-66 is replaced by valine (V157) in OXA-82.

#### 2.3.2. Aminoglycoside Resistance Genes

In 100% of *A. baumannii*, resistance to aminoglycosides was mediated by *aadA1*, *aacA4* and *armA* genes with the presence of the bi-functional gene *aac(6′)Ib-cr*. The *armA* gene confers resistance to gentamicin and amikacin, and it is generally plasmid-mediated. In four isolates we found the presence of other genes conferring resistance to aminoglycosides, such as *aph(3′)-IVa*, *aacA2*, *aadB*, *aac(3′)-Ic* and *aac(3)-Ia* (Table 1).

#### 2.3.3. Fluoroquinolone Resistance Genes

Resistance to fluoroquinolones is mediated by the *aac(6′)Ib-cr* gene, a bi-functional gene which confers resistance to both fluoroquinolones and aminoglycosides (Table 1).

#### 2.3.4. Other Antimicrobial Resistance Genes

The *mph(E)* and *msr(E)* (macrolide resistance), *sul1* (sulphonamide resistance), *tet(B)* (tetracycline resistance) and *catB8* (chloramphenicol resistance) genes were also detected in all *A. baumannii* isolates. The *strA/strB* genes, which confer resistance to streptomycin, were identified in 50% of the analyzed strains (Table 1).

### 2.4. Mobile Genetic Elements (MGEs)

A wide variety of sequence insertions (ISs) and transposon elements were identified in all analyzed strains (Table 1). In detail, transposon Tn*6207* was found in 3 *A. baumannii* isolates. Sequence insertion elements such as IS*Aba2* of *1308bp* length (3 isolates), IS*Aba13* of *1039* bp length (4 isolates), IS*Aba26* of 1318 bp length (4 isolates), IS*26* of 820 bp length (1 isolate), IS*Vsa3* of 977 bp (3 isolates), IS*Ec29* of 1325 bp (7 isolates), IS*6100* of 880 bp (4 isolates) and IS*17* of 1040 bp (4 isolates) were identified in the genome of *A. baumannii*.

### 2.5. Virulence Factors and Other Mechanisms of Resistance

Virulome analysis allowed to identify genes involved in biofilm (*bap* and *pgaABCD* locus) and pili (*csu*) formation. The *ompA* porin, lipid A synthesis (*lpxABCDLM* locus), biosynthesis of the LPS core (*lpsB*), serum resistance (*pbpG*), phospholipase C and D (*plc* and *plcD*), LysR-type transcriptional regulator as well as acinetobactin genes cluster (*bau*, *bas*, *bar*, *ent*) were found in all ST2 *A. baumannii* isolates (Table 2). The resistance-nodulation-cell division efflux pump genes (*RND: adeFGH*) were also found in the totality of the analyzed strains. The gene *hemO*, related to iron uptake, and the catalase gene (*katA*), which protects bacteria from superoxidants produced by leukocytes as a host defense mechanism, were also present in all sequenced strains.

## 3. Discussion

In this study, we used WGS to retrospectively investigate the resistance mechanisms in carbapenem- and colistin-resistant *A. baumannii* isolates in COVID-19 patients admitted to the ICU of Spirito Santo Hospital, Pescara (Central Italy). Analysis of the sequenced genomes revealed that all isolates shared the same MLST sequence type (ST2), which is the representative ST type of global clone II [28]. To date, the international clone ST2 is the most dominant type globally detected in all *A. baumannii* genomes sequenced [29]. Several studies showed the dissemination of CR*Ab* isolates harboring the *bla*_OXA-23-like_ and belonging to ST2 from different countries [30]. As reported in previous studies, *bla*_OXA-23_ was the most widely reported gene, and *bla*_OXA-66_ was the most common variant of OXA enzymes [31]. In our strains, the OXA-23 class D carbapenemase was identified in association with OXA-66 or OXA-82. The OXA-66 and OXA-82, which differ each other by L167V substitution, belong to the OXA-51-like enzymes. The OXA-51 is intrinsically over-expressed and it is able to confer high resistance to carbapenems in *A. baumannii* isolates [32]. The increased antibiotic resistance in *A. baumannii* is largely due to the actions of mobile genetic elements, to the activation of intrinsic resistance mechanisms, such as the chromosomal β-lactamase *bla*_ADC_, and to the presence of efflux pumps [33]. In our study we found the presence of an AmpC enzyme, the ADC-25 cephalosporinase, and the *AdeFGH RND* efflux pumps in all *A. baumannii* strains. To our knowledge, the *AdeFGH RND* efflux pumps seem to play a major role in acquired resistance and may also be associated with carbapenem non-susceptibility in *A. baumannii* [34,35].

The pathogenicity of *A. baumannii* isolates analyzed in the present study is exacerbated by the presence of the *abaI*/*abaR* quorum sensing system which is involved in morphology, growth characteristics, biofilm formation, motility, resistance and virulence of these microorganisms [36]. In addition, in our *A. baumannii* we found two-component signal transduction system *BfmRS*, which seem to be implicated in the control of various virulence-related traits and acting as a global modulator of *A. baumannii* physiology [37,38]. It is well known that, *A. baumannii* showing a loss of *BfmS* results in a significant reduction of motility [39]. *A. baumannii* represents a serious problem for public health because of its intrinsic and acquired antimicrobial resistance determinants and pathogenicity factors. The main types of mobile elements involved in the capture and mobilization of ARGs in Gram-negative pathogens, including *A. baumannii*, is represented by gene cassettes, transposons and sequence insertions (ISs) [40]. These mobile structures could disseminate antibiotic resistance determinants between pathogens compromising antibiotic treatment [41,42]. In the present study, we found a wide variety of ISs belonging to different IS families such as IS3 (IS*Aba2*), IS4 (IS*Ec29*), IS5 (IS*17* and IS*Aba13*), IS6 (IS*26* and IS*6100*), IS30 (IS*Aba125*), IS91 (IS*Vsa3*) and IS256 (IS*Aba26*). Generally, two copies of the same or related ISs can move resistance genes as part of a composite transposon, and some of the ISs include a strong promoter that drives expression of the captured gene [41]. The IS*Aba1* was first identified in *A. baumannii* upstream of *bla*_OXA-23_, *bla*_OXA-51_, *bla*_OXA-58_ and *bla*_ADC_-genes, and it seems to be an important factor in the genetic plasticity of this microorganism [43,44]. The presence of IS*Aba1* upstream of *bla*_OXA-51-like_ genes might represent a real mechanism of carbapenem resistance [45]. Insertion of IS*Aba125* in the *bla*_OXA-23_ promoter sequence has been reported to be associated with overexpression of *bla*_OXA-23_, *bla*_OXA-51_ and carbapenem resistance phenotypes in *A. baumannii* [46]. In all analyzed strains we also found gene structures indicating the presence of the Tn*6207* transposon. Tn*6207* is a composite transposon containing a tetracycline efflux pump and its regulator genes *tetB* and aminoglycoside resistance genes *strB* and *strA*.

Respiratory viral infections, such as SARS-CoV-2, predispose patients to bacterial co-infections and secondary infections [47,48,49,50]. This represents a high risk for hospitalized patients. In some patients we have ascertained the co-presence of other microorganisms such as *K. pneumoniae*. Bloodstream CR*Ab* and *K. pneumoniae* remain one of the most common ICU-acquired infections [50,51,52].

## 4. Materials and Methods

### 4.1. Strains Selection and Identification

A total of 43 *A. baumannii* strains were isolated from bloodstream infection of 43 COVID-19 patients with evident clinical signs of bacterial sepsis. The patients were admitted to the ICU ward of Spirito Santo Hospital (Pescara, Central Italy). Overall, 32 (74%) patients were male and 11 (26%) were female, with an age ranging from 31 to 82 years old. These patients had comorbidities such as kidney disease, diabetes, hypertension, or heart disease, and in some of them a co-presence of *E. faecalis*, *S. marcescens* and *K. pneumoniae* was also ascertained. The blood sample collection was repeated with an interval of 2 days for each patient. All samples were transferred to clinical molecular laboratory and analyzed following methods according to Clinical and Laboratory Standards Institute (CLSI) guidelines [53]. One *A. baumannii* strain per patient was included in the study. The strains, selected from positive cultures, were grown in Cled agar plates (Oxoid, UK) at 37 °C, overnight, and identified by matrix-assisted laser desorption ionization–time of flight (MALDI-TOF) mass spectrometry (Bruker Daltonics, Billerica, MA, USA).

### 4.2. Antimicrobial Susceptibility

Antimicrobial susceptibility was determined by the Phoenix system (Becton, Dickinson and Company, Sparks, MD, USA) at Clinical Microbiology and Virology Unit, Spirito Santo Hospital, Pescara, Italy. The MICs for amikacin, gentamicin, ciprofloxacin, meropenem, colistin and trimethoprim–sulfamethoxazole association were also performed by conventional broth microdilution procedures in Mueller–Hinton broth (Biolife Italiana, Milan, Italy) supplemented with calcium and magnesium (CAMHB), using an inoculum of 5 × 10^5^ CFU/mL according to CLSI [54].

### 4.3. WGS

A total of eight representative *A. baumannii* clinical strains, named PE/1-PE/8, were included in the genome sequencing. Total DNA was extracted using MagMax Microbiome Ultra Nucleic Acid Isolation kit (Applied Biosystems and ThermoFisher Scientific, Monza, Italy), according to the manufacturer’s instructions. Library preparation was performed as previously reported [55,56]. Sequencing of the library was carried out on an Illumina MiSeq using a paired-end 600-cycle protocol with a read length of 300 bp. DRAGEN FastQC + MultiQC v3.6.3 (https://basespace.illumina.com/apps/10562553/DRAGEN-FastQC-MultiQC, access date: 31 January 2022) were used for quality control and sequences filtering. Paired-end reads were assembled with Velvet v1.2.10 (https://basespace.illumina.com/apps/8556549/Velvet-de-novo-Assembly, access date: 21 February 2022). Multi-locus sequence typing (MLST) on assembled *A. baumannii* genomes was performed utilizing the Pasteur scheme. This scheme includes the identification of seven internal housekeeping genes: citrate synthase (*gltA*), homologous recombination factor (*recA*), 60-kDa chaperonin (*cpn60*), elongation factor EF-G (*fusA*), CTP synthase (*pyrG*), 50S ribosomal protein L2 (*rplB*) and RNA polymerase subunit B (*rpoB*) [57]. ResFinder4.1 and MobileElementFinder 1.0.3 were used to detect acquired antimicrobial resistance genes and mobile genetic elements, respectively (http://www.genomicepidemiology.org, accessed on 28 February 2022). Virulence Factor Database (VFDB) was used for the detection of virulence genes (http://www.mgc.ac.cn/VFs/, accessed on 4 March 2022).

## 5. Conclusions

In the present study all *A. baumannii* were isolated from patients which developed a bloodstream infection. A detailed molecular characterization of resistomes, including ARGs and MGEs, and virulence factor analysis were performed on the eight ST2 *A. baumannii* clinical isolates. This WGS analysis remarks the importance to know the genetic background of clinical pathogens. The use of a rapid method, such as next-generation sequencing, to identify resistomes and virulomes in clinical settings could be useful for an effective antimicrobial stewardship program.

## Figures and Tables

**Table 1:** Antimicrobial resistance genes (ARGs) and mobile genetic elements (MGEs) in bloodstream (BSI) *A. baumannii* isolates.

Strain	Genome Size (bp)	MGEs	Β-Lactam Resistance	Aminoglycoside Resistance	Macrolide Resistance	Fluoroquinolone Resistance	Others
*A. baumannii* PE1 (ST2)	4,023,584	Tn*6207* IS*Ec29* IS*Aba26* IS*26* IS*Aba125*	*bla*_ADC-25_ *bla*_OXA-23_ *bla*_OXA-66_	*aadA1* *aacA4* *aph(3′)-VIa* *aac(6′)Ib-cr* *strB* *strA* *armA*	*mph(E)* *msr(E)*	*aac(6′)Ib-cr*	*sul1* *tet(B)* *catB8*
*A. baumannii* PE2 (ST2)	4,023,635	Tn*6207* IS*Aba125* IS*Aba26* IS*Vsa3*	*bla*_ADC-25_ *bla*_OXA-23_ *bla*_OXA-66_	*aadA1* *aacA4* *aph(3′)-VIa* *aac(6′)Ib-cr* *strB* *strA* *armA*	*mph(E)* *msr(E)*	*aac(6′)Ib-cr*	*sul1* *tet(B)* *catB8*
*A. baumannii* PE3 (ST2)	3,850,309	IS*Ec29* IS*Aba125* IS*Aba2* IS*Aba13* IS*17* IS*6100*	*bla*_ADC-25_ *bla*_OXA-23_ *bla*_OXA-82_	*aadA1* *aacA2* *aacA4* *aadB* *aph(3′)-Ic* *aac(3)-Ia* *armA*	*mph(E)* *msr(E)*	*aac(6′)Ib-cr*	*sul1* *catB8*
*A. baumannii* PE4 (ST2)	3,965,839	Tn*6207* IS*Vsa3* IS*Ec29* IS*Aba125* IS*Aba26*	*bla*_ADC-25_ *bla*_OXA-23_ *bla*_OXA-66_	*aadA1* *aacA4* *strB* *strA* *armA*	*mph(E)* *msr(E)*	*aac(6′)Ib-cr*	*sul1* *tet(B)* *catB8*
*A. baumannii* PE5 (ST2)	3,829,825	IS*Ec29* IS*Aba125* IS*Aba2* IS*Aba13* IS*17* IS*6100*	*bla*_ADC-25_ *bla*_OXA-23_ *bla*_OXA-82_	*aadA1* *aacA2* *aacA4* *aadB* *aph(3′)-VIa* *aph(3′)-Ic* *aac(3)-Ia* *armA*	*mph(E)* *msr(E)*	*aac(6′)Ib-cr*	*sul1* *catB8*
*A. baumannii* PE6 (ST2)	4,041,529	Tn*6207* IS*Vsa3* IS*Ec29* IS*Aba125* IS*Aba26*	*bla*_ADC-25_ *bla*_OXA-23_ *bla*_OXA-66_	*aadA1* *aacA4* *strB* *strA* *armA*	*mph(E)* *msr(E)*	*aac(6′)Ib-cr*	*sul1* *tet(B)* *catB8*
*A. baumannii* PE7 (ST2)	3,841,128	IS*Ec29* IS*Aba125* IS*Aba2* IS*Aba13* IS*17* IS*6100*	*bla*_ADC-25_ *bla*_OXA-23_ *bla*_OXA-82_	*aadA1* *aacA2* *aacA4* *aadB* *aph(3′)-VIa* *aph(3′)-Ic* *aac(3)-Ia* *armA*	*mph(E)* *msr(E)*	*aac(6′)Ib-cr*	*sul1* *catB8*
*A. baumannii* PE8 (ST2)	3,852,243	IS*Ec29* IS*Aba125* IS*Aba2* IS*Aba13* IS*17* IS*6100*	*bla*_ADC-25_ *bla*_OXA-23_ *bla*_OXA-82_	*aadA1* *aacA2* *aacA4* *aadB* *aph(3′)-Ic* *aac(3)-Ia* *armA*	*mph(E)* *msr(E)*	*aac(6′)Ib-cr*	*sul1* *catB8*

**Table 2:** Virulence factors of the eight *A. baumannii* analyzed in this study.

Category	Virulence Factors	Related Genes
Adherence	Outer membrane protein	*ompA*
Biofilm formation	AdeFGH efflux pump	*adeF; adeG; adeH*
	Biofilm-associated protein	*bap*
	Csu fimbriae	*csuA; csuB; csuC; csuD; csuE*
	Polysaccharide poly-N-acetylglucosamine	*pgaA; pgaB; pgaC; pgaD*
Enzyme	Phospholipase C	*plc*
	Phospholipase D	*plcD*
Immune evasion	LPS	*lpsB; lpxA; lpxB; lpxD; lpxL; lpxM*
Iron uptake	Acinetobactin	*barA; barB; basA; basB; basC; basD; basF; basG; basH; basI; basJ; bauA; bauB; bauC; bauD; bauE; bauF; entE;*
	Heme utilization	*hemO*
Regulation	Quorum sensing	*abaI; abaR*
	Two-component system (BfmRS)	*bfmR; bfmS*
Serum resistance	PbpG (Penicillin-binding protein)	*pbpG*
Stress adaption	Catalase	*katA*

## Data Availability

Not applicable.
